# Exploring the Interest and Intention to Use Long-Acting Injectable PrEP (LAI-PrEP) Among Gay, Bisexual and Other Men Who Have Sex with Men (GBMSM) in the Netherlands

**DOI:** 10.1007/s10461-026-05038-0

**Published:** 2026-01-24

**Authors:** Johann Kolstee, Haoyi Wang, Hanne Zimmermann, Melanie Schroeder, Ama Appiah, Supriya Sarkar, Ana Milinkovic, Kai Jonas

**Affiliations:** 1 Faculty of Psychology and Neuroscience - Maastricht, Maastricht University, Universiteitssingel 40, PO Box 616, 6200 MD Maastricht, The Netherlands; 2 ViiV Healthcare Ltd, Brentford, UK; 3 ViiV Healthcare, Durham, NC USA; 4 Chelsea and Westminster Hospital, London, UK

**Keywords:** HIV, Prevention, PrEP, Men who have sex with men

## Abstract

Long-acting injectable PrEP (LAI-PrEP) provides opportunities to expand HIV prevention coverage in the Netherlands. Understanding which Dutch gay, bisexual and other men who have sex with men (GBMSM) are interested in using LAI-PrEP when available can support HIV prevention efforts. Survey data was collected online via the PROTECT survey from 1,447 GBMSM in the Netherlands between October 2023 and March 2024. Multivariable logistic analysis was conducted to determine variables associated with GBMSM who intend to use LAI-PrEP when available. Interest and intention to use LAI-PrEP among Dutch GBMSM is high (76.1% and 73.9%) and among those currently on oral PrEP even higher (80.8% and 79.5%). GBMSM who intend to use LAI-PrEP have more sexual partners in the past 6 months (11–50 partners (aOR = 3.24; 95%CI: 1.54–6.85), 51–100 partners (aOR = 3.10; 95% CI: 1.20 to 8.30), more than 150 partners (aOR = 3.86; 95% CI: 1.74 to 8.67)), engage more in chemsex (aOR = 1.41; 95% CI: 1.00 to 2.01), are less likely to fear LAI-PrEP side effects (aOR = 0.83; 95% CI: 0.74 to 0.94;) or injection pain (aOR = 0.68; 95% CI: 0.61 to 0.75) and more likely to believe they are at risk of acquiring HIV (aOR = 1.23; 95% CI: 1.05 to 1.45). If made available in the Netherlands, many GBMSM are likely to commence using LAI-PrEP, particularly current oral PrEP users or discontinued users. Sexually active Dutch GBMSM, who are already protecting themselves, may be the first group to use LAI-PrEP.

**Supplementary Information:**

The online version contains supplementary material available at 10.1007/s10461-026-05038-0.

## Resumen

La PrEP inyectable de acción prolongada (PrEP-IAP) ofrece oportunidades para ampliar la cobertura de la prevención del VIH en los Países Bajos. Comprender cuales hombres gay, bisexuales y otros hombres que tienen sexo con hombres (GBHSH) holandeses están interesados en usar la PrEP-IAP cuando esté disponible puede respaldar las iniciativas de prevención del VIH. Los datos de la encuesta se recopilaron en línea a través de la encuesta PROTECT de 1447 GBHSH en los Países Bajos entre octubre de 2023 y marzo de 2024. Se realizó un análisis logístico multivariable para determinar las variables asociadas con los GBHSH que tienen intención de usar la PrEP-IAP cuando esté disponible. El interés y la intención de usar la PrEP-LAI entre los GBHSH holandeses es alto (76,1% y 73,9%), y entre quienes actualmente toman la PrEP oral es aún mayor (80,8% y 79,5%). Los GBHSH que tienen intención de utilizar PrEP-IAP tienen más parejas sexuales en los últimos 6 meses (11–50 parejas (ORa = 3,24; IC del 95%: 1,54 − 6,85), 51–100 parejas (ORa = 3,10; IC del 95%: 1,20 a 8,30), más de 150 parejas (ORa = 3,86; IC del 95%: 1,74 a 8,67)), participan más en chemsex (ORa = 1,41; IC del 95%: 1,00 a 2,01), tienen menos probabilidades de temer los efectos secundarios de la PrEP-IAP (ORa = 0,83; IC del 95%: 0,74 a 0,94) o el dolor de la inyección (ORa = 0,68; IC del 95%: 0,61 a 0,75) y es más probable que crean que están en riesgo de contraer el VIH (ORa = 1,23; IC del 95%: 1,05 a 1,45). Si se hace disponible en los Países Bajos, es probable que muchos GBHSH comiencen a usar PrEP-IAP, en particular quienes ya usan la PrEP oral, o la han dejado de usar. Los GBHSH holandeses sexualmente activos, que ya se protegen, podrían ser el primer grupo en usar PrEP-IAP.

## Introduction

Increasing the range of HIV prevention strategies is required to end the HIV epidemic in the Netherlands and globally [1, 2]. Pre-exposure prophylaxis for HIV (PrEP) is effective at preventing HIV transmission [3–6]. PrEP use along with condoms and treatment as prevention must continue to be promoted to curb infections in the Netherlands [1, 2]. The potential of PrEP has not yet been fully realized in the Netherlands, with people who are eligible and willing to take it still facing challenges with access to PrEP (e.g., lack of full subsidization by the Dutch health system) and others who already take oral PrEP struggling with taking it effectively, either daily or on demand [7–10]. Providing alternative ways in which PrEP can be taken, such as via long-acting injections, has the potential to provide more effective prevention coverage for people who do not use oral PrEP regimens as indicated [11, 12]. These new modalities also have the potential to engage a new group of people, currently not using oral PrEP, with effective HIV prevention strategies [13, 14]. They can provide greater convenience, as they require less frequent administration. However, these new ways of receiving PrEP are not yet available in the Netherlands.

Oral PrEP has been formally available since 2016 in the Netherlands via a prescription from a doctor, but not subsidized, and only free of charge for participants in the AMPrEP study, a prospective, open-label study of gay, bisexual and other men who have sex with men (GBMSM) and transgender people, to evaluate the feasibility and uptake of daily and event-driven oral PrEP [15, 16]. With the expansion of the availability of PrEP via general practitioners in 2018 and via the local public health departments (Gemeentelike Gezondheidsdienst), in 2019 through the national PrEP pilot program, many GBMSM have commenced oral PrEP (8,500 slots) [10, 17]. Attitudes towards oral PrEP in the Netherlands have improved since PrEP was made formally available, currently key affected populations believe that PrEP is effective and many people, particularly GBMSM, are interested in PrEP or are already taking PrEP [18–21].

Some current oral PrEP users may be struggling with adherence in the Netherlands [22]. It has been suggested that this group may also benefit greatly from alternative, often more convenient, ways of taking PrEP, and could be a target for long-acting injectable PrEP (LAI-PrEP) implementation in the Netherlands [22, 23]. Since PrEP implementation commenced in the Netherlands, some people have discontinued PrEP. Research has indicated that people stop taking PrEP for many reasons, including the inconvenience of taking daily pills, cost, changes in their sexual behavior and worries about side effects [7]. Former PrEP users may also benefit from novel PrEP modalities such as LAI-PrEP [24, 25].

LAI-PrEP, specifically Cabotegravir, which is delivered via an intermuscular injection every two months, has been approved for use in the European Union by the European Medicines Agency (EMA) in 2023. However, it is still not available for use as a prevention tool in the Netherlands. It was approved in the United States of America (USA) for use by the Federal Drug Agency in 2021 and has been available for use there since 2022. In the USA, uptake has been slow, due to cost and access issues [26]. In this analysis, we sought to understand the proportion of GBMSM in the Netherlands who were interested and who intend to use LAI-PrEP, if it becomes available. We also sought to explore the determinants of intention to use LAI-PrEP among GBMSM in the Netherlands.

## Methods

### Study Design and Participants

The data utilized in this analysis was collected as part of the “*Understanding pre-exposure prophylaxis modalities for HIV prevention in European communities”*, (PROTECT) survey. The survey was cross-sectional and only available online. Qualtrics XM^®^ software was used to collect data. Survey responses were collected from October 2023 to April 2024. The survey was promoted to GBMSM, and to transgender and non-binary people, via gay dating apps (Grindr and Hornet) and via social media advertisements (Facebook and Instagram). Participants could also reach the survey via the survey website (https://protect-study.eu/*).* The survey was made available in 22 languages, mainly European languages and some key migrant languages, and data collection occurred in 20 European countries. This analysis has focused on data collected from GBMSM residing in the Netherlands. To be eligible to participate they had to be 18 years of age or older. Consent was provided online prior to commencing the survey. Ethical approval for this research was provided by the Ethics Review Committee Psychology and Neuroscience (ERCPN), of Maastricht University (OZL_262_08_01_2023_S21).

### Measures

The measures utilized in the PROTECT survey have been described previously [27] and incorporate a large set of variables. Variables used in this analysis, and their categories, are presented in Tables 1 and 2. The variables chosen aligned with the PROTECT survey aims and also with variables known to be associated with use of oral PrEP [27, 28].

**Table 1:** Participant characteristics

Variable	Response option	All participants		Current PrEP users (*N* = 697)		PrEP inexperienced (*N* = 646)		Discontinued PrEP users (*N* = 104)		Test statistics*
		*N* (median)	% (IQR)	*N* (median)	% (IQR)	*N* (median)	% (IQR)	*N* (median)	% (IQR)	
Age (years)	18–24	119	8.2	30	4.3	73	11.3	16	15.4	*χ² (12) = 52.8*, *p* < 0.001
	25–29	163	11.3	63	9	78	12.1	22	21.2	
	30–39	386	26.7	203	29.1	157	24.3	26	25	
	40–49	315	21.8	165	23.7	138	21.4	12	11.5	
	50–59	259	17.9	139	19.9	104	16.1	16	15.4	
	60–69	113	7.8	52	7.5	54	8.4	7	6.7	
	70+	92	6.4	45	6.5	42	6.5	5	4.8	
Sexual orientation	Gay	1212	83.8	626	89.8	490	75.9	96	92.3	*χ² (4) = 67.7*, *p* < 0.001
	Bisexual	190	13.1	49	7	137	21.2	4	3.8	
	Other	45	3.1	22	3.2	19	2.9	4	3.8	
Education level	I do not have a high school diploma	18	1.2	3	0.4	12	1.9	3	2.9	*χ² (8) = 28.1*, *p* < 0.001
	Secondary education (high school or equivalent)	385	26.6	164	23.5	198	30.7	23	22.1	
	Bachelor degree (university or equivalent)	562	38.8	270	38.7	247	38.2	45	43.3	
	Master degree (university or equivalent)	417	28.8	229	32.9	164	25.4	24	23.1	
	PhD / Doctorate	65	4.5	31	4.4	25	3.9	9	8.7	
Employment	Employed	1058	73.1	553	79.3	432	66.9	73	70.2	*χ² (8) = 39.8*, *p* < 0.001
	Other	158	10.9	61	8.8	88	13.6	9	8.7	
	Retired/Medical leave	74	5.1	35	5	36	5.6	3	2.9	
	Student	124	8.6	36	5.2	71	11	17	16.3	
	Unemployed	33	2.3	12	1.7	19	2.9	2	1.9	
Comfort on current income	Living comfortably on present income	708	48.9	360	51.6	303	46.9	45	43.3	*χ² (8) = 22.9*, *p* = 0.003
	Living really comfortably on present income	176	12.2	91	13.1	77	11.9	8	7.7	
	Neither comfortable nor struggling on present income	373	25.8	170	24.4	166	25.7	37	35.6	
	Really struggling on present income	55	3.8	15	2.2	32	5	8	7.7	
	Struggling on present income	135	9.3	61	8.8	68	10.5	6	5.8	
Size of place of residence	A very big city or town (a million or more people)	133	9.2	92	13.2	32	5	9	8.7	*χ² (8) = 93.4*, *p* < 0.001
	A big city or town (500,000–999,999 people)	401	27.7	241	34.6	129	20	31	29.8	
	A medium-sized city or town (100,000–499,999 people)	446	30.8	198	28.4	213	33	35	33.7	
	A small city or town (10,000–99,999 people)	297	20.5	112	16.1	172	26.6	13	12.5	
	A village / the countryside (less than 10,000 people)	170	11.7	54	7.7	100	15.5	16	15.4	
Migrant	First Generation migrant	289	20	164	23.5	102	15.8	23	22.1	*χ² (4) = 15.2*,*p* = 0.004
	Local	1020	70.5	464	66.6	487	75.4	69	66.3	
	Second Generation migrant	138	9.5	69	9.9	57	8.8	12	11.5	
Relationship status	Single	309	21.4	142	20.4	145	22.4	22	21.2	*χ² (6) = 81.7*, *p* < 0.001
	Dating	379	26.2	178	25.5	170	26.3	31	29.8	
	In a monoganous Relationship	341	23.6	110	15.8	207	32	24	23.1	
	In an open/polyamorous relationship	418	28.9	267	38.3	124	19.2	27	26	
Vaginal sex in past 6 months	No	1298	89.7	643	92.3	558	86.4	97	93.3	*χ² (2) = 14.1*, *p* < 0.001
	Yes	149	10.3	54	7.7	88	13.6	7	6.7	
Number of sex partners	0	40	2.8	2	0.3	36	5.6	2	1.9	*χ² (12) = 244.7*, *p* < 0.001
	1	87	6	11	1.6	68	10.5	8	7.7	
	(2–10)	498	34.4	155	22.2	308	47.7	35	33.7	
	(11–50)	525	36.3	338	48.5	150	23.2	37	35.6	
	51–100	73	5	55	7.9	11	1.7	7	6.7	
	101–150	17	1.2	13	1.9	4	0.6	0	0	
	150+	207	14.3	123	17.6	69	10.7	37	35.6	
Position preference in anal sex	Top only	206	14.2	86	12.3	106	16.4	14	13.5	*χ² (6) = 57.1*, *p* < 0.001
	Bottom only	180	12.4	66	9.5	101	15.6	13	12.5	
	No anal sex	33	2.3	2	0.3	31	4.8	0	0	
	Versatile	1028	71	543	77.9	408	63.2	77	74	
Substance use in past 6 months to enhance sex	No	827	57.2	304	43.6	464	71.8	59	56.7	*χ² (2) = 109*, *p* < 0.001
	Yes	620	42.8	393	56.4	182	28.2	45	43.3	
Chemsex in past 6 month	No	1063	73.5	423	60.7	560	86.7	80	76.9	*χ² (2) = 116.9*, *p* < 0.001
	Yes	384	26.5	274	39.3	86	13.3	24	23.1	
PEP use ever	No	1313	90.7	588	84.4	646	100	79	76	*χ² (2) = 126.7*, *p* < 0.001
	Yes	134	9.3	109	15.6	0	0	25	24	
Condomless anal sex	No	196	13.5	35	5	146	22.6	15	14.4	*χ² (2) = 88.5*, *p* < 0.001
	Yes	1251	86.5	662	95	500	77.4	89	85.6	
Condomless anal sex with PLHIV partner	No	1421	98.2	675	96.8	642	99.4	104	100	*χ² (2) = 14.3*, *p* < 0.001
	Yes	26	1.8	22	3.2	4	0.6	0	0	
Unprotected sex	Higher risk	839	58	250	35.9	500	77.4	89	85.6	*χ² (2) = 272.4*, *p* < 0.001
	Lower risk	608	42	447	64.1	146	22.6	15	14.4	
Payment given for sex ever	No	1195	82.6	576	82.6	532	82.4	87	83.7	*χ² (2) = 0.1*, *0.947*
	Yes	252	17.4	121	17.4	114	17.6	17	16.3	
Payment received for sex ever	No	1280	88.5	622	89.2	582	90.1	76	73.1	*χ² (2) = 26.2*, *p* < 0.001
	Yes	167	11.5	75	10.8	64	9.9	28	26.9	
HIV testing frequency	Frequently testing	634	43.8	539	77.3	60	9.3	35	33.7	*χ² (8) = 759.3*, *p* < 0.001
	Every six months	321	22.2	120	17.2	161	24.9	40	38.5	
	Less than once per year	165	11.4	5	0.7	147	22.8	13	12.5	
	Once per year	194	13.4	31	4.4	148	22.9	15	14.4	
	Never	133	9.2	2	0.3	130	20.1	1	1	
STI testing frequency	Frequently testing	618	42.7	526	75.5	57	8.8	35	33.7	*χ² (8) = 744.0*, *p* < 0.001
	Every six months	327	22.6	129	18.5	158	24.5	40	38.5	
	Less than once per year	188	13	10	1.4	165	25.5	13	12.5	
	Once per year	183	12.6	31	4.4	138	21.4	14	13.5	
	Never	131	9.1	1	0.1	128	19.8	2	1.9	
PrEP use history	Current	697	48.2	697	100	NA	NA	NA	NA	
	Former	104	7.2	0	0	NA	NA	104	100	
	Inexperienced	646	44.6	0	0	646	100	NA	NA	
Fear of needles		1	(1–2)	1	(1–2)	1	(1–2)	1	(1–2)	*0.194*
Fear of injection pain		1	(1–3)	1	(1–3)	1	(1–3)	1	(1–2)	*0.246*
Fear of LAI-PrEP side effects		3	(2–4)	3	(2–4)	3	(2.25–4.25)	3	(1.75–4.75)	*p* < 0.001
Perception that one will acquire HIV		2	(1–3)	2	(1–2)	2	(1–3)	2	(2–3)	*p* < 0.001
Worry about HIV		2	(1.5–2.5)	2	(1–2)	2	(2–2)	2	(2–3)	*p* < 0.001

* Chi-square tests were applied to test the differences between GBMSM with different oral prep use status

**Table 2:** Interest and intention to use LAI-PrEP

Variable	Response options	All participants		Current PrEP users		PrEP Inexperienced		Discontinued PrEP users		p-value
		n	%	n	%	n	%	n	%	
Interest to use LAI-PrEP	No	112	7.7	34	4.9	73	11.3	5	4.8	*p* < 0.001
	Undecided	234	16.2	100	14.3	120	18.6	14	13.5	
	Yes	1101	76.1	563	80.8	453	70.1	85	81.7	
Interest to use LAI-PrEP (Binary)	No	346	23.9	134	19.2	453	70.1	19	18.3	*p* < 0.001
	Yes	1101	76.1	563	80.8	193	29.9	85	81.7	
Intention to use LAI-PrEP	Extremely likely	628	43.4	358	51.4	211	32.7	59	56.7	*p* < 0.001
	Somewhat likely	442	30.5	196	28.1	221	34.2	25	24	
	Neither likely nor unlikely	182	12.6	73	10.5	97	15	12	11.5	
	Somewhat unlikely	91	6.3	42	6	44	6.8	5	4.8	
	Extremely unlikely	104	7.2	28	4	73	11.3	3	2	
Intention to use LAI-PrEP (Binary)	No	377	26.1	143	20.5	214	33.1	20	19.2	*p* < 0.001
	Yes	1070	73.9	554	79.5	432	66.9	84	80.8	

Use of oral PrEP was assessed with the item, “*Are you currently using oral PrEP?”*, with response categories including “yes” or “no”. Participants who selected “no” were then also asked, “*Have you ever used oral PrEP in the past?”*, with response categories including “Yes, I used oral PrEP in the last six month”, “Yes, I used oral PrEP but more than six months ago”, or “No, I have never used oral PrEP”. Participants who reported never having used PrEP were classified as *“PrEP inexperienced”*, participants who had previously used PrEP but were currently not using it were classified as *“Former PrEP users”.*

LAI-PrEP interest was assessed with the item “*After knowing more about LAI-PrEP*,* are you interested in using LAI-PrEP if it is available to you?”*, with response categories including yes, no and undecided. Intention to use LAI-PrEP was assessed using a 1-item assessment. Participants were asked to rate the following item, “*How likely are you to start using LAI-PrEP every 2 months if it is available to you?”* by selecting a response on a 5-point Likert scale from 1 (Extremely unlikely) to 5 (Extremely likely).

Both interest in LAI-PrEP and intention to use LAI-PrEP were measured. We included a measure to explore intention, as it goes beyond interest and asks participants to consider if they would take action to utilise this tool, not merely if they have an interest in using it, and therefore is a closer indication of future behaviour [29].

The fear of injection pain, fear of needles, and fear of LAI-PrEP side effects, were assessed using items that participants responded to by selecting a response on a 5-point Likert scale from 1 (strongly disagree) to 5 (strongly agree). The perception that one will acquire HIV was assessed using a 1-item assessment that participants responded to by selecting a response on a 5- point Likert scale from 1 (extremely unlikely) to 5 (extremely likely). Worry about acquiring HIV was assessed using a 1-item assessment that participants responded to by selecting a response on a 5-point Likert scale from 1 (never) to 5 (always).

### Statistical Analyses

We included data from GBMSM in the PROTECT survey who were residing in the Netherlands in this analysis. Participants who were living with HIV were excluded as were transgender and non-binary people, data on these groups will be reported separately. Descriptive data, including data on interest and intention to use LAI-PrEP, were presented for the whole sample and also separately for current PrEP users, PrEP inexperienced and former PrEP users. Chi-square tests were applied to test the demographic differences between GBMSM with different oral prep use status. To explore the group of GBMSM who intend to use LAI-PrEP, univariable logistic regression was used to test for statistical significance, with the dependent variable constructed as a binary variable (intention to use LAI-PrEP vs. non-intention). Participants were classified as intending to use LAI-PrEP if they indicated that they were likely or extremely likely to start using LAI-PrEP every 2 months if it was available to them, on the scale, described above, used to measure LAI-PrEP intention. Variables found to be statistically significant at the univariable level (*p* < 0.05) were included in a multivariable logistic regression. This was conducted firstly on the whole sample to determine which variables were significantly associated with participants who indicated they intended to use LAI-PrEP. We repeated this approach with current oral PrEP users. This was done as current PrEP users are a potential target group for LAI-PrEP promotion, once it becomes available, as they are already familiar with PrEP use. We reported adjusted odds ratios and 95% confidence intervals (CI). The statistical significance was set at *p* < 0.05 and all analyses were conducted using R (version 4.3.2).

## Results

### Participant Characteristics

Our sample for this analysis was comprised of 1,447 survey responses from GBMSM in the Netherlands. Table 1 provides further information about our sample’s characteristics.

### Demographics

The median age of our participants was 41 (Interquartile range (IQR) 31–52) years. Most men reported that they were gay (83.8%) or bisexual (13.1%). The majority of men had completed at least a bachelor’s degree (72.1%) and were employed (73.1%). Only a small proportion of men reported struggling or really struggling on their present income (13.1%). About two thirds (67.7%) of our sample lived in at least a medium sized city (100,000–499,999 people) or larger. A significant minority of men were born outside of the Netherlands (20%) or had at least one parent who was born outside of the Netherlands (9.5%).

### Sexual Behaviour

Most men had more than one sexual partner in the past six months (91.2%) and most reported some versatility in the sexual position they take during anal sex (71%). Many GBMSM in our sample reported some substance use to facilitate or enhance their sexual activity in the past six months (42.8%) and around a quarter (26.5%) reported chemsex (the use of one or more of the following drugs (ecstasy/MDMA, GHB, cocaine, amphetamine, crystal methamphetamine, mephedrone or ketamine) to enhance sexual experiences). Some men in our sample reported that at some point in their lives they had paid for sex (17.4%), a smaller proportion of men ported that at some point in their lives they had been paid for sex (11.5%).

### Oral PrEP Use

Approximately half of the GBMSM in our sample reported current PrEP use (*n* = 697, 48.2%), many were PrEP inexperienced (*n* = 646, 44.6%), and a much smaller group reported having previously taken PrEP but having discontinued use (*n* = 104, 7.2%). The characteristics of participants are reported in Table 1.

Several important differences exist between current PrEP users and the rest of the GBMSM in our sample who have either previously taken PrEP and currently are not, or who are PrEP inexperienced. Compared to the overall sample, current PrEP users in our sample tended to be slightly older (χ² [12] = 52.8, *p* < 0.001). They were also more likely to identify as gay (89.8%) than PrEP inexperienced participants (75.9%) (χ² [4] = 67.7, *p* < 0.001), more educated than PrEP inexperienced or discontinued users (χ² [8] = 28.1, *p* < 0.001), and more likely to be employed (χ² [8] = 39.8, *p* < 0.001). Current PrEP users also tend to live in a bigger city (χ² [8] = 93.4, *p* < 0.001), and were more likely to be in an open relationship (χ² [6] = 81.7, *p* < 0.001). Current PrEP users also were more likely to have more than 10 sex partners in the past 6 months (χ² [12] = 244.7, *p* < 0.001), have chemsex (χ² [2] = 116.9, *p* < 0.001), and test for HIV (χ² [8] = 759.3, *p* < 0.001), and STIs (χ² [8] = 744.0, *p* < 0.001), frequently.

#### Interest and Intention to Use LAI-PrEP

The majority of GBMSM (*n* = 1101, 76.1%) in our sample from the Netherlands reported interest in using LAI-PrEP when it becomes available for use. Current PrEP users (*n* = 563, 80%) and discontinued PrEP users (*n* = 85, 81.7%) were significantly more interested (χ² [2] = 22.9, *p* < 0.001) than PrEP inexperienced participants (*n* = 453, 70.1%). Please see Table 2 for more information.

The majority of GBMSM (*n* = 1070, 73.9%) also reported the intention (either “somewhat likely” or “extremely likely”) to use LAI-PrEP when it becomes available for use in the Netherlands. Again, current PrEP users (*n* = 554, 79.5%) and discontinued PrEP users (*n* = 84, 80.8%) were significantly more likely (χ² [2] = 30.4, *p* < 0.001) to report that they intend to use PrEP when available in the Netherlands than PrEP inexperienced participants (*n* = 432, 66.9%).

#### Barriers to LAI-PrEP Use

The median score (MS) across this sample of GBMSM was low on scales exploring fears about LAI-PrEP, such as the fear towards needles (MS = 1, IQR ([1]– [2])), and fear of pain (MS = 1, IQR [1–3]). This indicates that most participants either strongly disagreed [1] or somewhat disagreed [2] that they had fears of needles or of injection pain. Participants in this sample scored somewhat higher on the fear of side effects scale (MS = 3, IQR [2–4]). The MS were also low for a scale asking participants if they thought they were likely to acquire HIV (MS = 2, IQR [1–3]), and for a scale asking participants how frequently they worry about getting HIV (MS = 2, IQR (1.5–2.5)) - no differences were observed in the results for current PrEP users on this scale.

#### Determinants of Intention to Use LAI-PrEP

Several variables were associated with GBMSM who reported they intended to use LAI-PrEP when available in the Netherlands. The results of a multivariable analysis are presented in Table 3.

**Table 3:** Results of multivariable analysis (all GBMSM)

Variable	Response option	Adjusted Odds Ratio	CI_low	CI_high	*p*.value
Size of place of residence	A very big city or town (a million or more people)	Reference			
	A big city or town (500,000–999,999 people)	0.71	0.40	1.23	0.230
	A medium-sized city or town (100,000–499,999 people)	0.54	0.30	0.93	0.030
	A small city or town (10,000–99,999 people)	0.81	0.45	1.44	0.490
	A village / the countryside (less than 10,000 people)	0.59	0.31	1.09	0.095
Condomless anal sex	Yes	1.40	0.96	2.03	0.080
	No	Reference			
Chemsex in past 6 month	Yes	1.41	1.00	2.01	0.051
	No	Reference			
PEP use ever	Yes	1.39	0.83	2.45	0.230
	No	Reference			
Number of sex partners	0	Reference			
	1	0.94	0.42	2.12	0.880
	101–150	1.87	0.49	8.28	0.376
	11–50	3.24	1.54	6.85	0.002
	150+	3.86	1.74	8.67	0.001
	2–10	1.83	0.90	3.78	0.097
	51–100	3.10	1.20	8.30	0.021
Position preference in anal sex	Top	Reference			
	Bottom	0.96	0.59	1.56	0.869
	Not anal sex	0.37	0.15	0.85	0.021
	Versatile	1.09	0.75	1.56	0.651
Worry about HIV	Likert scale	1.40	1.16	1.70	0.001
Perception that one will acquire HIV	Likert scale	1.23	1.05	1.45	0.013
Fear of injection pain	Likert scale	0.68	0.61	0.75	0.000
Fear of LAI-PrEP side effects	Likert scale	0.83	0.74	0.94	0.002
PrEP use history	Current	Reference			
	Former	1.03	0.58	1.89	0.923
	Inexperienced	0.82	0.59	1.14	0.229

GBMSM in our sample who intended to use LAI-PrEP were more likely to have had higher numbers of sexual partners in the past 6 months (11–50 partners, *aOR = 3.24; 95%CI: 1.54–6.85*,*p* = 0.002), 51–100 partners (*aOR = 3.10; 95% CI: 1.20–8.30*,*p* = 0.021), more than 150 partners (*aOR = 3.86; 95% CI: 1.74–8.67*,*p* = 0.001). They were more likely to engage in chemsex (*aOR = 1.41; 95% CI: 1.00- 2.01*,*p* = 0.051). These men were also more likely to think they were likely to acquire HIV (*aOR = 1.23; 95% CI: 1.05–1.45*,*p* = 0.013) and to worry frequently about acquiring HIV (*aOR = 1.40; 95% CI: 1.16–1.70*,*p* = 0.001).

GBMSM in our sample who intended to use LAI-PrEP were less likely to be living in a medium sized city (*aOR = 0.54; 95% CI: 0.30–0.93*,*p* = 0.030) and report not having any anal sex (*aOR = 0.37; 95% CI: 0.15–0.85*,*p* = 0.021). GBMSM in our sample who intended to use LAI-PrEP were less likely to report fear of injection pain (*aOR = 0.68; 95% CI: 0.61–0.75*,*p* < 0.001) or side effects from LAI-PrEP use (*aOR = 0.83; 95% CI: 0.74–0.94*,*p* = 0.002).

The results of the multivariable analysis on current PrEP users alone are presented in Table 4 (for the results of the univariable analysis please see supplementary materials). This multivariable analysis sought to determine variables associated with GBMSM, currently taking PrEP, who reported they intended to use LAI-PrEP when available in the Netherlands.

**Table 4:** Results of multivariable analysis (current PrEP users only)

Variable	Response option	Adjusted Odds Ratio	CI_low	CI_high	*p*.value
Migrant	Local	0.57	0.32	0.98	0.046
	First Generation migrant	Reference			
	Second Generation migrant	0.64	0.29	1.43	0.263
Size of place of residence	A very big city or town (a million or more people)	Reference			
	A big city or town (500,000–999,999 people)	0.56	0.23	1.21	0.157
	A medium-sized city or town (100,000–499,999 people)	0.34	0.14	0.74	0.009
	A small city or town (10,000–99,999 people)	0.78	0.30	1.93	0.602
	A village / the countryside (less than 10,000 people)	0.49	0.18	1.32	0.163
Chemsex in past 6 month	Yes	1.67	1.10	2.58	0.018
	No	Reference			
HIV testing frequency	Frequently testing (less than every six months)	Reference			
	Every six months	1.30	0.75	2.32	0.362
	Less than once per year	1.76E + 06	0.000	NA	0.981
	Never	1.19E-07	NA	4.70E + 62	0.988
	Once per year	0.37	0.16	0.91	0.026
Worry about HIV	Likert scale	1.34	0.98	1.89	0.076
Perception that one will acquire HIV	Likert scale	1.46	1.11	1.95	0.008
Fear of injection pain	Likert scale	0.71	0.61	0.82	0.000
Fear of LAI-PrEP side effects	Likert scale	0.77	0.64	0.93	0.006
PrEP use regimen	Daily	Reference			
	Mixed use	0.95	0.51	1.84	0.870
	On-demand	0.74	0.47	1.17	0.198

GBMSM who reported current PrEP use and who intended to use LAI-PrEP were more likely to engage in chemsex (*aOR = 1.67; 95% CI: 1.10–2.58*,*p* = 0.018) and to think they were likely to acquire HIV (*aOR = 1.46; 95% CI: 1.11–1.95*,*p* = 0.008). GBMSM who reported current PrEP use and who intended to use LAI-PrEP were less likely to be a local with no migrant background (*aOR = 0.57; 95% CI: 0.32–0.98*,*p* = 0.046) and to be living in a medium sized city (*aOR = 0.34; 95% CI: 0.14–0.74*,*p* = 0.009). They were also less likely to report testing for HIV only once per year (*aOR = 0.37; 95% CI: 0.16–0.90*,*p* = 0.026), less likely to report fearing injection pain (*aOR = 0.71; 95% CI: 0.61–0.82*,*p* < 0.001) or side effects from LAI-PrEP use (*aOR = 0.77; 95% CI: 0.64–0.93*,*p* = 0.006).

## Discussion

Our analysis has demonstrated that GBMSM in the Netherlands are very interested in alternatives to oral PrEP. LAI-PrEP, if made available, could meet the HIV prevention needs of approximately three-quarters (73.9%) of this group. Interest in LAI-PrEP was found to be slightly higher than participant’s intent to actually receive the medication. GBMSM who intend to use LAI-PrEP are more sexually active and engage more in chemsex, they fear less often the potential LAI-PrEP side effects or injection pain and believe more often that they are at risk of acquiring HIV. Many GBMSM in the Netherlands are already taking oral PrEP, with about half of our sample (48.2%) reporting current PrEP use, this is somewhat higher than other research on this population, although from somewhat earlier in PrEP implementation in the Netherlands [30, 31]. The interest and intent to use LAI-PrEP among this group of GBMSM in the Netherlands was slightly higher than among the overall sample. Similarly, to the overall sample, GBMSM who are current PrEP users who intend to use LAI-PrEP are more sexually adventurous, less likely to fear the potential LAI-PrEP side effects or injection pain, and believe they are at more risk of acquiring HIV. Additionally, among GBMSM living in the Netherlands who are current PrEP users, having a lower likelihood to intend to use LAI-PrEP was also associated with being a local/non-migrant and testing for HIV infrequently.

The results presented in this analysis reinforce the fact that PrEP has become a core HIV prevention strategy for GBMSM in the Netherlands [16, 17, 20, 32, 33]. A high proportion of this group are aware of PrEP and are already utilizing oral PrEP either daily or on demand. An even higher proportion of GBMSM in the Netherlands group intend to use LAI-PrEP when available and accessible, a novel finding. GBMSM have a history of picking up HIV prevention strategies and being proactive about their sexual health, having adapted to and lived through the HIV epidemic for over 40 years [34, 35]. Their enthusiastic response to new PrEP modalities is not surprising in this context. Switching to having to remember to protect oneself from HIV at two-month intervals instead of daily is undoubtedly liberating for many [36].

Our results indicate that while not commonly reported, fear of LAI-PrEP side effects and of injection pain do remain important considerations for some people and should be considered and addressed in efforts to rollout LAI-PrEP. This mirrors findings from other jurisdictions [24, 37]. GBMSM who are sexually active and adventurous and who are conscious and aware of their HIV risk have traditionally been the targets of health promotion work [35]. These groups are often easier to reach via community organizations or health services and could present a first wave of adopters for LAI-PrEP. While current oral PrEP users are slightly more interested in LAI-PrEP, the high interest and intention among GBMSM with different PrEP use histories (e.g. PrEP inexperienced, former users and current users) indicates that a widespread and less targeted implementation campaign may be most effective. Some current oral PrEP users may switch to LAI-PrEP and some PrEP inexperienced individuals could directly start PrEP via injections and never take oral PrEP [28]. Health systems must promote a diversity of HIV prevention options for all. This allows individuals to choose which strategy is right for them at different times in their sexual lives. This “toolbox approach” has been shown to be effective at engaging GBMSM [38–40]. Seasons of risk are also known to exist across the lifespan during which the HIV prevention strategy that a person utilizes changes [41–43]. Giving people choices that are accessible and affordable supports people moving between different strategies as their own risk profile changes [44].

LAI-PrEP and other HIV prevention strategies such as patches and implants may be made available in the Netherlands and across Europe over the coming years. The price of LAI-PrEP is not yet clear, and governments and health systems may choose to only provide these new forms of protection to certain groups to provide value for money for the health system. Our results indicate that among GBMSM in the Netherlands a substantial opportunity exists to engage this group with HIV prevention via injectable PrEP. Despite their PrEP use history, age and education, many GBMSM in the Netherlands are ready to use this new technology with the support of their healthcare providers. Although GBMSM in our sample with a PrEP use history were significantly more likely to intend to use LAI-PrEP, PrEP use history was not significant in our multivariable regression analysis, indicating that efforts to engage GBMSM should involve the entire population, and could include different messaging for groups with different PrEP use histories.

Our research has some limitations. As LAI-PrEP is not yet available in the Netherlands questions about its potential future use are still hypothetical at this stage. Our research was cross-sectional in nature and so it did not track changes in the intention to use LAI-PrEP over time. Our research was cross-sectional in nature and is the first data available on interest and intention to use LAI-PrEP among GBMSM in Europe. It did not track changes in the intention to use LAI-PrEP over time. While this was not the intention of this research project, it could be beneficial in future to track a cohort of individuals overtime to understand changes in attitudes, knowledge and beliefs towards HIV prevention strategies, including LAI-PrEP. The focus of our enquiry was to understand the interest and intention to use LAI-PrEP as a bi-monthly injectable (Cabotegravir), as this is the only LAI-PrEP product that is currently approved for use in Europe by the EMA. Other LAI-PrEP products may also soon be approved for use in Europe, such as Lenacapavir, which is administered every six months [45]. Our survey was not able to capture the differences that may exist in the interest and intention of different LAI-PrEP products or options. Furthermore, as it was unknown at the time of data collection what the price of LAI-PrEP Cabotegravir would be set at in the Netherlands, we were unable to incorporate the issue of actual cost into the exploration of interest and intention to use this product. We would expect that the cost of using LAI-PrEP could present a substantial barrier to its use, if set too high. It could be useful to explore both the topic of preferences between different LAI-PrEP products and the issue of cost in future research, once there is more information available on alternative products and on the pricing of Cabotegravir.

In conclusion, LAI-PrEP, when made available could help to provide improved HIV prevention coverage among GBMSM in the Netherlands. High interest and intention to use LAI-PrEP among GBMSM in the Netherlands, and experience as a community with oral PrEP, provide a pathway for LAI-PrEP roll-out.

## Supplementary Information

Below is the link to the electronic supplementary material.

Supplementary Material 1
