# Supplementary material for: Exploring the Interest and Intention to Use Long-Acting Injectable PrEP (LAI-PrEP) Among Gay, Bisexual and Other Men Who Have Sex with Men (GBMSM) in the Netherlands
Source: AIDS Behav. 2026 Jan 24;30(7):2129–39. doi: 10.1007/s10461-026-05038-0 (PMC13400490; doi:10.1007/s10461-026-05038-0)
Supplement: Supplementary file 1 — Supplementary Material 1 [file 10461_2026_5038_MOESM1_ESM.docx]

**SUPPLEMENTARY MATERIALS**

**Table 5 - Results of univariable analysis (all GBMSM)**

| **Variable** | **Response Option** | **OddsRatio** | **PValue** | **LowerCI** | **UpperCI** |
| --- | --- | --- | --- | --- | --- |
| **Age (years)** | 25-29 | 1.48 | 0.13 | 0.89 | 2.47 |
|  | 30-39 | 1.60 | 0.04 | 1.03 | 2.47 |
|  | 40-49 | 1.78 | 0.01 | 1.13 | 2.80 |
|  | 50-59 | 1.84 | 0.01 | 1.14 | 2.94 |
|  | 60-69 | 1.43 | 0.20 | 0.82 | 2.51 |
|  | 70+ | 2.17 | 0.02 | 1.17 | 4.14 |
| **Education level** | Secondary education (high school or equivalent) | 1.97 | 0.17 | 0.71 | 5.15 |
|  | Bachelor degree (university or equivalent) | 1.67 | 0.30 | 0.61 | 4.32 |
|  | Master degree (university or equivalent) | 2.07 | 0.14 | 0.74 | 5.41 |
|  | PhD / Doctorate | 1.16 | 0.78 | 0.38 | 3.37 |
| **Employment** | Other | 0.80 | 0.23 | 0.55 | 1.16 |
|  | Retired/Medical leave | 0.83 | 0.48 | 0.50 | 1.42 |
|  | Student | 0.66 | 0.04 | 0.45 | 1.00 |
|  | Unemployed | 1.02 | 0.96 | 0.48 | 2.45 |
| **Comfort on current income** | Living really comfortably on present income | 0.67 | 0.03 | 0.47 | 0.95 |
|  | Neither comfortable nor struggling on present income | 1.01 | 0.94 | 0.76 | 1.35 |
|  | Really struggling on present income | 1.09 | 0.80 | 0.58 | 2.15 |
|  | Struggling on present income | 1.00 | 0.99 | 0.66 | 1.54 |
| **Migrant** | Local | 0.74 | 0.06 | 0.54 | 1.01 |
|  | Second Generation migrant | 0.98 | 0.94 | 0.61 | 1.61 |
| **Size of place of residence** | A big city or town (500,000-999,999 people) | 0.68 | 0.15 | 0.40 | 1.13 |
|  | A medium-sized city or town (100,000-499,999 people) | 0.46 | 0.00 | 0.27 | 0.74 |
|  | A small city or town (10,000-99,999 people) | 0.60 | 0.06 | 0.35 | 1.00 |
|  | A village / the countryside (less than 10,000 people) | 0.40 | 0.00 | 0.23 | 0.70 |
| **Relationship status** | Dating | 1.64 | 0.01 | 1.16 | 2.32 |
|  | In a monogamous Relationship | 0.92 | 0.61 | 0.66 | 1.28 |
|  | In an open/polyamorous relationship | 1.44 | 0.03 | 1.03 | 2.02 |
| **Sexual orientation** | Bisexual | 0.77 | 0.12 | 0.55 | 1.08 |
|  | Other | 0.74 | 0.37 | 0.40 | 1.46 |
| **Condomless anal sex** | Yes | 1.41 | 0.04 | 1.02 | 1.95 |
| **Condomless anal sex with PLHIV partner** | Yes | 0.79 | 0.58 | 0.35 | 1.94 |
| **Unprotected sex** | Lower risk | 1.02 | 0.86 | 0.81 | 1.30 |
| **Chemsex in past 6 month** | Yes | 2.19 | 0.00 | 1.63 | 2.98 |
| **Substance use in past 6 months to enhance sex** | Yes | 1.79 | 0.00 | 1.40 | 2.30 |
| **PEP use ever** | Yes | 2.13 | 0.00 | 1.33 | 3.57 |
| **HIV testing frequency** | Every six months | 0.86 | 0.36 | 0.62 | 1.19 |
|  | Less than once per year | 0.41 | 0.00 | 0.28 | 0.59 |
|  | Never | 0.36 | 0.00 | 0.24 | 0.53 |
|  | Once per year | 0.59 | 0.00 | 0.41 | 0.86 |
| **Number of sex partners** | 1 | 1.04 | 0.92 | 0.49 | 2.22 |
|  | 101-150 | 3.97 | 0.03 | 1.18 | 16.08 |
|  | 11-50 | 5.19 | 0.00 | 2.69 | 10.16 |
|  | 150+ | 6.44 | 0.00 | 3.14 | 13.48 |
|  | 2-10 | 2.63 | 0.00 | 1.37 | 5.10 |
|  | 51-100 | 5.64 | 0.00 | 2.42 | 13.75 |
| **Position preference in anal sex** | Bottom only | 0.91 | 0.68 | 0.59 | 1.41 |
|  | No anal sex | 0.26 | 0.00 | 0.12 | 0.55 |
|  | Versatile | 1.30 | 0.13 | 0.92 | 1.80 |
| **STI testing frequency** | Every six months | 0.82 | 0.23 | 0.59 | 1.14 |
|  | Less than once per year | 0.38 | 0.00 | 0.27 | 0.55 |
|  | Never | 0.42 | 0.00 | 0.28 | 0.63 |
|  | Once per year | 0.58 | 0.00 | 0.40 | 0.84 |
| **Payment given for sex ever** | Yes | 1.22 | 0.23 | 0.89 | 1.69 |
| **Payment received for sex ever** | Yes | 1.38 | 0.11 | 0.94 | 2.06 |
| **Vaginal sex in past 6 months** | Yes | 0.92 | 0.67 | 0.63 | 1.36 |
| **PrEP use history** | Former | 1.08 | 0.76 | 0.66 | 1.87 |
|  | Naïve | 0.52 | 0.00 | 0.41 | 0.67 |
| **Worry about HIV** |  | 1.45 | 0.00 | 1.24 | 1.71 |
| **Perception that one will acquire HIV** |  | 1.39 | 0.00 | 1.21 | 1.59 |
| **Fear of injection pain** |  | 0.69 | 0.00 | 0.63 | 0.76 |
| **Fear of needles** |  | 0.77 | 0.00 | 0.71 | 0.85 |
| **Fear of LAI-PrEP side effects** |  | 0.75 | 0.00 | 0.68 | 0.84 |

**Table 6 - Results of univariable analysis (current PrEP users only)**

| **Variable** | **Response Option** | **OddsRatio** | **PValue** | **LowerCI** | **UpperCI** |
| --- | --- | --- | --- | --- | --- |
| **Age (years)** | 25-29 | 0.70 | 0.49 | 0.25 | 1.87 |
|  | 30-39 | 1.17 | 0.74 | 0.44 | 2.78 |
|  | 40-49 | 1.26 | 0.62 | 0.47 | 3.08 |
|  | 50-59 | 1.39 | 0.50 | 0.51 | 3.47 |
|  | 60-69 | 1.01 | 0.98 | 0.34 | 2.90 |
|  | 70+ | 1.98 | 0.27 | 0.59 | 6.85 |
| **Education level** | Secondary education (high school or equivalent) | 3.07 | 0.37 | 0.14 | 33.27 |
|  | Bachelor degree (university or equivalent) | 1.71 | 0.66 | 0.08 | 18.18 |
|  | Master degree (university or equivalent) | 1.89 | 0.61 | 0.09 | 20.08 |
|  | PhD / Doctorate | 1.05 | 0.97 | 0.05 | 12.28 |
| **Employment** | Other | 1.04 | 0.91 | 0.55 | 2.10 |
|  | Retired/Medical leave | 0.63 | 0.24 | 0.30 | 1.42 |
|  | Student | 0.89 | 0.78 | 0.41 | 2.14 |
|  | Unemployed | 2.79 | 0.33 | 0.54 | 51.33 |
| **Comfort on current income** | Living really comfortably on present income | 0.72 | 0.23 | 0.43 | 1.25 |
|  | Neither comfortable nor struggling on present income | 1.12 | 0.64 | 0.71 | 1.79 |
|  | Really struggling on present income | 1.68 | 0.50 | 0.45 | 10.91 |
|  | Struggling on present income | 1.18 | 0.65 | 0.60 | 2.48 |
| **Migrant** | Local | 0.55 | 0.02 | 0.33 | 0.89 |
|  | Second Generation migrant | 0.64 | 0.23 | 0.31 | 1.36 |
| **Size of place of residence** | A big city or town (500,000-999,999 people) | 0.46 | 0.04 | 0.20 | 0.94 |
|  | A medium-sized city or town (100,000-499,999 people) | 0.27 | 0.00 | 0.12 | 0.56 |
|  | A small city or town (10,000-99,999 people) | 0.57 | 0.19 | 0.23 | 1.30 |
|  | A village / the countryside (less than 10,000 people) | 0.31 | 0.01 | 0.12 | 0.77 |
| **Relationship status** | Dating | 1.38 | 0.25 | 0.79 | 2.40 |
|  | In a monogamous Relationship | 0.94 | 0.84 | 0.52 | 1.70 |
|  | In an open/polyamorous relationship | 1.15 | 0.59 | 0.70 | 1.87 |
| **Sexual orientation** | Bisexual | 0.90 | 0.76 | 0.46 | 1.89 |
|  | Other | 1.64 | 0.43 | 0.55 | 7.07 |
| **Condomless anal sex** | Yes | 1.36 | 0.44 | 0.59 | 2.88 |
| **Condomless anal sex with PLHIV partner** | Yes | 0.68 | 0.43 | 0.27 | 1.92 |
| **Unprotected sex** | Lower risk | 0.64 | 0.03 | 0.42 | 0.95 |
| **Chemsex in past 6 month** | Yes | 1.60 | 0.02 | 1.08 | 2.38 |
| **Substance use in past 6 months to enhance sex** | Yes | 1.27 | 0.21 | 0.87 | 1.83 |
| **PEP use ever** | Yes | 1.48 | 0.17 | 0.87 | 2.65 |
| **HIV testing frequency** | Every six months | 1.20 | 0.50 | 0.73 | 2.05 |
|  | Less than once per year | 1459538.61 | 0.98 | 0.00 | NA |
|  | Never | 0.00 | 0.99 | NA | NA |
|  | Once per year | 0.46 | 0.05 | 0.22 | 1.02 |
| **Number of sex partners** | 1 | 1.20 | 0.91 | 0.04 | 35.97 |
|  | 101-150 | 3.33 | 0.44 | 0.11 | 104.48 |
|  | 11-50 | 4.20 | 0.31 | 0.16 | 107.15 |
|  | 150+ | 7.20 | 0.17 | 0.28 | 188.66 |
|  | 2-10 | 2.60 | 0.50 | 0.10 | 66.85 |
|  | 51-100 | 4.00 | 0.34 | 0.15 | 106.69 |
| **Position preference in anal sex** | Bottom only | 0.66 | 0.28 | 0.31 | 1.40 |
|  | No anal sex | 0.00 | 0.98 | NA | NA |
|  | Versatile | 1.02 | 0.96 | 0.56 | 1.76 |
| **STI testing frequency** | Every six months | 0.94 | 0.81 | 0.59 | 1.54 |
|  | Less than once per year | 0.58 | 0.44 | 0.16 | 2.73 |
|  | Never | 0.00 | 0.98 | NA | NA |
|  | Once per year | 0.86 | 0.72 | 0.38 | 2.20 |
| **Payment given for sex ever** | Yes | 1.05 | 0.84 | 0.65 | 1.75 |
| **Payment received for sex ever** | Yes | 1.26 | 0.47 | 0.69 | 2.46 |
| **Vaginal sex in past 6 months** | Yes | 1.15 | 0.71 | 0.58 | 2.47 |
| **PrEP regimen** | Mixed use | 1.15 | 0.64 | 0.65 | 2.13 |
|  | On demand | 0.83 | 0.36 | 0.56 | 1.24 |
| **Worry about HIV** |  | 1.54 | 0.00 | 1.19 | 2.03 |
| **Perception that one will acquire HIV** |  | 1.52 | 0.00 | 1.20 | 1.95 |
| **Fear of injection pain** |  | 0.71 | 0.00 | 0.62 | 0.81 |
| **Fear of needles** |  | 0.77 | 0.00 | 0.67 | 0.89 |
| **Fear of LAI-PrEP side effects** |  | 0.78 | 0.00 | 0.66 | 0.91 |
